# Distilling the evidence for GLP-1 receptor agonists in alcohol use disorder

**DOI:** 10.1186/s13722-025-00638-y

**Published:** 2025-12-24

**Authors:** Eden Y. Bernstein, Joseph P. Schacht

**Affiliations:** 1 Division of Hospital Medicine, Department of Medicine, University of Colorado School of Medicine, 12401 East 17th Avenue, 4th floor, Aurora, CO 80045 USA; 2 Department of Psychiatry, University of Colorado School of Medicine, Aurora, CO USA

Globally, unhealthy alcohol use contributes to 2.6 million annual deaths [1]. Alcohol use disorder (AUD) can be effectively treated with medications and behavioral treatments. However, while FDA-approved medications like naltrexone and acamprosate have demonstrated efficacy, their effect sizes are modest, and many patients do not benefit from them [2]. Novel and effective treatments for AUD are urgently needed to mitigate alcohol-related harms. Glucagon-like peptide-1 receptor agonists (GLP-1 RAs), widely used for the treatment of diabetes and obesity, have emerged as a potential therapeutic for AUD with promising early findings.

In their systematic review and meta-analysis, Sinha and Ghosal synthesized available evidence on the effects of GLP-1RAs on alcohol-related outcomes following rigorous best practices, including pre-registration, adherence to reporting guidelines, and author contact for incorporation of missing data [3]. This review is timely given the rapid pace of research in this field and increasing cultural relevance of GLP-1 RAs. The authors identified six observational studies and three randomized trials that assessed alcohol-related outcomes across several GLP-1 RAs. In a pooled meta-analysis of observational studies, they identified an association between GLP-1 RA use and a reduction of alcohol-related events, which the authors defined as AUD incidence, recurrence, hospitalizations related to alcohol or other substances, or acute alcohol intoxication (HR 0.64, 95% CI 0.59–0.69). These observational studies relied on methods such as propensity score matching to account for differences between treatment groups which may be related to receipt of treatment and confound the association with the outcome (i.e., confounding by indication) [4]. While these results are promising, such study designs cannot account for differences between treatment groups which are not available in the data, known as unmeasured confounding. Clinical trials that achieve comparability by allocating treatment groups randomly across participants are needed to corroborate these encouraging observational findings.

Fortunately, Sinha and Ghosal also meta-analyzed three randomized trials in their review. In contrast to the analysis of observational studies, the pooled meta-analysis of these three trials showed a non-significant association between GLP-1 RAs and alcohol-related outcomes, including total alcohol consumption, drinks per drinking day, alcohol craving, and heavy drinking days (SMD − 0.24, 95% CI -0.70, 0.23). While these results may temper enthusiasm, a closer look also provides reasons for optimism. First, the trials by Hendershot [5] and Probst [6] both found statistically significant reductions in alcohol-related outcomes when comparing GLP-1 RAs (semaglutide and dulaglutide, respectively) with placebo. In the non-significant trial of exenatide by Klausen [7], both treatment groups also received cognitive behavioral therapy, an evidence-based treatment for AUD that may have attenuated the observed effect of the drug. This study also had a high attrition rate (less than 50% of patients in either group participated in the 26-week follow-up), limiting statistical power to detect effects. Second, the discrepant findings across trials may reflect differences in efficacy across types of GLP-1 RAs. For example, semaglutide, which showed the largest effect for alcohol-related outcomes among trials in this review, also exhibits greater effects for weight loss compared to dulaglutide and exenatide [8]. 

While at present there is insufficient evidence to conclusively support the efficacy of GLP-1 RAs for AUD, we may not have to wait long for definitive answers. Sinha and Ghosal identified eight ongoing trials which will advance our understanding of this topic. Of them, four are trials of oral or injectable semaglutide, two of which have been completed but have not yet reported results. Notably, two ongoing trials are investigating the dual glucose-dependent insulinotropic polypeptide (GIP) and GLP-1 RA tirzepatide, which demonstrates an even greater effect than semaglutide for weight loss outcomes [8] and demonstrated the largest effect on alcohol-related outcomes among types of GLP-1 RAs in Sinha and Ghosal’s subgroup analysis of observational studies.

If efficacy of GLP-1 RAs for AUD is established in these ongoing trials, many research questions will remain that will be of interest to *Addiction Science & Clinical Practice* and the broader field of AUD research. First, we will need comparative efficacy trials of GLP-1 RAs against existing medications for AUD like naltrexone. The trial by Hendershot et al. reported effect sizes of semaglutide on alcohol consumption larger than those associated with naltrexone or acamprosate, but head-to-head trials will be needed to inform recommendations for first-line treatments. Second, it will be important to describe heterogeneous treatment effects including differences across AUD phenotypes (e.g., reward-motivated drinking) [9] and biologic characteristics. For example, an exploratory analysis by Klausen, et al. found that exenatide reduced alcohol intake among the subgroup of patients with BMI > 30 kg/m [7]. Third, optimal dosing will need to be determined. Patients in the observational trials reviewed by Sinha and Ghosal were presumably taking GLP-1 RA doses recommended for obesity or glycemic control (e.g., for injectable semaglutide, 2-2.4 mg/week), but in the Hendershot et al. trial, a substantially lower injectable semaglutide dose of 0.5 mg/week had a large effect size for reduction in drinking [5]. Fourth, recommended duration of treatment should be established to determine whether discontinuation of GLP-1 RAs will result in return to pre-treatment drinking patterns, as has been observed in animal studies [10]. Fifth, severe underutilization of existing evidence-based AUD treatments [11] and disparities noted in access to GLP-1 RAs for diabetes and obesity [12] suggest there will be a need for novel hybrid effectiveness-implementation trials to increase equitable access to GLP-1 RAs among patients with AUD [13]. 

Sinha and Ghosal summarize potentially promising early evidence for the efficacy of GLP-1 RAs for AUD. If ongoing trials confirm efficacy, these medications could provide a sorely needed addition to the available AUD treatment options and mark a paradigm shift in the pharmacologic treatment of AUD.
